# Electrocardiogram Parameters That Affect the Success Rate of Radiofrequency Ablation in Patients with Outflow Tract Ventricular Premature Complexes

**DOI:** 10.1155/2022/8160144

**Published:** 2022-07-22

**Authors:** Zhen Ye, Zhe Xu, Kezeng Gong, Xuehai Chen, Weiwei Wang, Jianhua Chen, Lianglong Chen, Feilong Zhang

**Affiliations:** ^1^Department of Rheumatology, Ningde Municipal Hospital of Ningde Normal University, Fujian 352000, China; ^2^Department of Cardiology, Fujian Medical University Union Hospital, Fuzhou 350001, China; ^3^Fujian Heart Medical Center, Fuzhou 350001, China; ^4^Fujian Institute of Coronary Heart Disease, Fuzhou 350001, China; ^5^Fujian Institute of Geriatrics, Fuzhou 350001, China

## Abstract

**Objectives:**

The objectives of this study are to assess the efficacy of radiofrequency catheter ablation (RFCA) in patients with outflow tract (OT) ventricular premature complexes (VPCs) and to explore the electrocardiographic (ECG) features of initially successful procedures.

**Methods:**

Based on the outcome of ablation, 154 consecutive patients with OT-VPCs who underwent RFCA from January 2017 to December 2019 were divided into two groups. The rate of successful procedures and the ECG features were analyzed and compared between the two groups.

**Results:**

The highest success rate was found in patients with VPCs from the right ventricular outflow tract (RVOT), and the lowest success rate was evident among patients with complexes from both the RVOT and the left ventricular OT (LVOT). The patients with successful procedures (136) reflected a lower pseudo delta wave ratio (16.2% vs. 44.4%, *P* < 0.01), a smaller R-wave amplitude in lead V1 (V1) (0.23 ± 0.24 mV vs. 0.35 ± 0.44 mV, *P* < 0.05), shorter intrinsicoid deflection time in lead V2 (V2) (44.00 ± 18.33 ms vs. 57.41 ± 20.67 ms, *P* < 0.01), a shorter RS duration in V2 (93.67 ± 21.33 ms vs. 106.93 ± 18.76 ms, P < 0.01), and smaller R/S-waveratios in V2. Furthermore, multivariate analysis demonstrated that RS duration in V2 was above 109.17 ms and R/S ratio in V2 was above 0.28, forecasting a failed procedure.

**Conclusions:**

The ECG predictors of failed ablation were characterized by RS duration and R/S ratio in V2.

## 1. Introduction

Ventricular premature complexes (VPCs) are one of the most common arrhythmias in patients with and without structural heart disease and are typically considered benign. However, a higher frequency of VPCs was associated with a decrease in left ventricular ejection fraction (LVEF), an increase in incident congestive heart failure, and increased mortality within a large, prospective, multicenter study conducted by the American Institute of Cardiovascular Health  [1]. Therefore, VPCs may present the reversible risk of cardiomyopathy, which has also been extensively described in the literature at home and abroad in recent years. Ventricular premature complex-induced cardiomyopathies are classified as acquired dilative cardiomyopathy. The condition requires the exclusion of other underlying disorders and observations of the left ventricle (LV), as well as functional improvement of left ventricle following successful arrhythmia control for diagnosis. However, the pathogenesis of VPC-induced cardiomyopathy remains unclear. There is support for the notion that long-term repeated ventricular asynchronous contraction may lead to changes in the cardiac structure and function; concurrently, it is also believed that a shorter coupling interval between VPCs results in the reduction of effective cardiac output and the decline of cardiac function  [2].

Accordingly, the early treatment of patients with VPCs is important. Treatment approaches include drug therapies, e.g., drugs to control ventricular rate and antiarrhythmic drugs. Another approach is the use of radiofrequency catheter ablation (RFCA). Although antiarrhythmic drug therapies have played an important role in the initial management of VPCs, by targeting the arrhythmogenic foci within the myocardium, RFCA provides an alternative, safe, and potentially curative strategy for drug-refractory cases [3, 4]. The advancement of electroanatomical mapping and ablation technology has led to a high success rate for RFCA of approximately 80%–90% [5, 6], with variable recurrence and failure rates being reported. However, the electrocardiographic (ECG) features of failed ablation with regard to idiopathic VPCs have not been clarified.

In the present study, we aimed to identify the ECG features of failed VPC ablation to provide useful information for preoperative communication with patients, the decision to effect an ablation strategy and to help guide future research for establishing a better ablation method.

## 2. Materials and Method

### 2.1. Study Population

From January 2017 to December 2019, a total of 154 consecutive patients in our hospital undergoing RFCA with frequent outflow tract VPCs (OT-VPCs), confirmed by electrophysiological examination, were retrospectively recruited into our study. The sample included 42 men (27.3%) and 112 women (72.7%) with a median age of 50 years (11–75 years); the median duration of enrollment in the study was 12 months. The patients' baseline characteristics are shown in Table 1. All patients had a complete medical history, and preoperative examinations included an ECG, echocardiography, and 24-hour Holter monitoring.

### 2.2. Inclusion Criteria

All of the patients included in our study had ECG features that showed a left bundle branch block or right bundle branch block, as well as high amplitude (R)-waves in leads II, III, and augmented vector foot (aVF). Concurrently, the density of VPCs, which was derived by Holter monitoring, was greater than 10,000 beats/24 h or greater than 10% of the total number of beats. Free from antiarrhythmic drugs, all patients underwent electrophysiological examination and RFCA by the same team of experienced electrophysiologists, and the electrophysiological examination confirmed that the VPCs originated from the outflow tract (OT).

### 2.3. Exclusion Criteria

We excluded patients with severe diseases, such as severe cardiopulmonary insufficiency, hypohepatia, renal dysfunction, coagulation disorders, electrolyte disturbances, and serious infection. Second, we excluded patients who had viral myocarditis, myocardial infarction, or a stroke during the preceding six months, as well as patients with a malignancy who had a life expectancy shorter than one year. Patients with complicated psychological conditions who were unable to manage the treatment were also excluded. Finally, we ruled out patients with severe deformity of the thoracic cage and a lack of relevant data (e.g., ECG patterns).

### 2.4. Definition and Measurement of the Electrocardiogram Criteria

**Figure 1:**
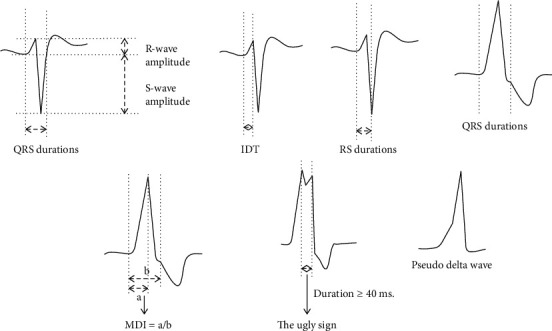
The measurement of the electrocardiogram criteria.

Prior to conducting the RFCA, a 12-lead surface ECG was performed using the conventional technique at a paper speed of 25 mm/s and with a sensitivity of 10 mm/mV. The following ECG characteristics were reviewed (see Figure 1 for example).

#### 2.4.1. The QRS Complex Durations

The QRS complex durations (ms) were defined as the interval measured from the earliest ventricular activation (or from the stimulation artifact) to the offset of the QRS complex in leads II, III, aVF, and V2. We measured three QRS durations continuously in each lead, and their arithmetic mean values were calculated.

#### 2.4.2. The Q, R, and S-Wave Amplitudes

The R-wave amplitude (mV) was defined as the height measured from an isoelectric line to the peak of the QRS complex in leads II, III, aVF, V1, and V2. We also continuously measured three R-wave amplitudes in each lead and calculated their arithmetic mean values as the final measurements. Similarly, we obtained the S-wave amplitude in lead V2 and the Q-wave amplitude in the augmented vector left (aVL), and augmented vector right (aVR) leads.

#### 2.4.3. Intrinsicoid Deflection Time

The intrinsicoid deflection time (IDT) was defined as the time from the QRS onset to the peak QRS deflection (or to the peak of the R-wave or to the second peak of the notch if the R-wave notched) in lead V2. The IDT values were taken as the mean of three initial IDT measurements in lead V2.

#### 2.4.4. The RS Durations

The RS durations were defined as the interval measured from the QRS onset to the trough of the S-wave in lead V2. The R and S-wave durations of three QRS waves were measured continuously, and the resulting arithmetic mean value was taken as the final result.

#### 2.4.5. The R-Wave Ratio of Leads III and II

The R-wave ratio of leads III and II was defined as the R-wave amplitude in lead III divided by the R-wave amplitude in lead II.

#### 2.4.6. The R and S-Wave Ratios in Lead V2

The R and S-wave ratios in lead V2 were defined as the R-wave amplitude divided by the S-wave amplitude in lead V2.

#### 2.4.7. The Augmented Vector Left and Augmented Vector Right Q-Wave Ratios

The Q-wave ratio of the aVL/aVR leads was defined as the Q-wave amplitude in lead aVL divided by the Q-wave amplitude in lead aVR.

#### 2.4.8. Maximum Deflection Index

The maximum deflection index (MDI) was defined as the inferior lead presenting the tallest R-wave; this was derived by dividing the time from the QRS onset to the peak QRS deflection by the total QRS duration.

#### 2.4.9. Corrected QT Interval

The corrected QT (QTc) interval is the QT interval corrected according to heart rate, which is an indicator that reflects the depolarization and repolarization of the heart. Its calculation method in this study was as follows: (Bazett's formula): QTc = QT/(RR^0.5), where RR is the standardized heart rate value (RR = 60/heart rate).

#### 2.4.10. Retrograde P-Wave

The atrial depolarization wave caused by a retrograde impulse into the atrium is called the retrograde P-wave because the conduction direction of its excitement is in opposition to the direction of the sinus P-wave.

#### 2.4.11. Interpolated Ventricular Premature Complexes

Interpolated VPCs refer to the appearance of a broad malformed QRS wave group between two normal sinus beats and without the compensatory interval.

#### 2.4.12. The Ugly Sign

The ugly sign was considered to be an obvious setback or as flat segments in a broadly malformed QRS complex when the duration of the surge was ≥40 ms.

#### 2.4.13. The Pseudo Delta Wave

The pseudo delta wave was defined when there was no smooth curve between the beginning and the deflection of the QRS complex.

### 2.5. Echocardiography

Echocardiographic testing was recorded before the ablation procedure to assess the heart structure and function. All patients were placed in the left decubitus position and were required to maintain a steady breathing rate. The standard parasternal long axis section of the LV, five-chamber apical view, and four-chamber standard apical view were adopted for observation to fulfill the need for three cycles of measuring. The measurements were as follows: (1) left ventricular end-diastolic diameter (LVEDD), left ventricular end-systolic diameter (LVESD), LVEF, left ventricular late diastolic filling peak flow rate, and left ventricular early diastolic filling peak flow rate (PVE), as well as the adjusted value (E') of ∗∗∗∗, i.e., E/E' and E/A.

### 2.6. Electrophysiological Study and Radiofrequency Catheter Ablation

Written informed consent forms for inclusion in the study were obtained from all patients. Antiarrhythmic agents were discontinued for a minimum of five half-lives before conducting RFCA. We performed a standardized electrophysiological study and RFCA for all patients with intraoperative monitoring for arterial blood pressure and blood oxygen saturation.

In the operative process, all patients were placed in the supine position with routine disinfection, towel-laying, and 1% lidocaine local anesthesia. The ablation catheter was retrogradely advanced to the RVOT, coronary sinus, or aortic root area and positioned to the ablation site via right femoral vein/femoral artery access. A three-dimensional mapping system (CARTO3) was used for mapping in all patients. The ablation site was identified by the exact or closest QRS pace map matched to the tachycardia or ectopy QRS and/or the earliest endocardial activation preceding the QRS. Ablation was performed using a Thermocool (TC) cold saline infusion catheter. If the VPC disappeared within 10 s, radiofrequency energy was delivered at a pulse duration of 60–90 s for each point in a temperature-controlled mode at 43°C and an electrosurgical unit of 30 W. Successful ablation was defined as the complete elimination of predominant VPCs with and without isoprenaline infusion at least 30 min after ablation. If detailed repeat mapping and ablation in the left and right OTs were performed, the VPCs did not decrease or had acute recurrences after stopping radiofrequency energy, the result was considered a failed ablation. Finally, the catheter and sheath tube were withdrawn, and the wound was compressed to stop bleeding using sterile bandages. Following RFCA, the patient was returned to the ward.

### 2.7. Case Division

The 154 enrolled patients were divided into two groups according to the outcomes of RFCA. The group reflecting a very successful procedure included 136 patients (34 males and 102 females) with a mean age of (48.28 ± 13.20) years, a median disease course of 12 months, and LVEF of 65.13% ± 5.86%. The group representing failed procedures included 18 cases (8 males and 10 females) with an average age of 56.00 ± 26.00 years, a median course of nine months, and LVEF of 64.65% ± 8.75%.

Acute or initial successful ablation was defined as the complete disappearance of a targeted VPC, on and off isoproterenol, immediately after radiofrequency ablation, and no recurrence for 30-min post-ablation.

### 2.8. Follow-Up

All patients were followed up regularly in the outpatient clinic or by telephone or another form of communication. Recurrence was defined as presence of targeted VPC on 12-lead electrocardiogram or 24-hour Holter monitoring off antiarrhythmic drugs during follow-up. Based on the relevant symptoms, the ECG patterns, performed using a 12-lead electrocardiogram or 24-hour Holter monitoring, recurrence after RFCA, the success rate of reoperation, and the occurrence of acute or long-term complications in patients were recorded.

### 2.9. Statistical Analysis

The SPSS Statistics 25.0 software program was used to conduct statistical analysis in our study. According to the sample size, a Kolmogorov–Smirnov or Shapiro–Wilk test was conducted to analyze the normality of the measurement data. If the distribution conformed to the normal distribution, continuous variables were described as mean ± standard deviation (X ± SD), the comparison of which was effected using the Student's *t*-test. If the distribution did not conform to the normal distribution, the measurement data were expressed as the median ± quad interval and compared using the Mann–Whitney *U* test. Categorical variables were described as frequencies and percentages (*n*%), and the difference was compared using the chi-square test. Fisher's exact test was used when the sample size was smaller than 5 in a given cell. The association between selected parameters and a failed RFCA was studied using binary logistic regression analysis. The variables that were selected for testing in multivariate analysis were those reflecting a *P* < 0.05 in the univariate models. Receiver-operating characteristic (ROC) analysis for the identification of failed ablation was performed to calculate the sensitivity, specificity, the areas under the ROC curve, and the optimal cut-off value. In all tests, the criterion for statistical significance was a two-sided *P* < 0.05.

## 3. Results

### 3.1. Patient Characteristics

In this study, 154 consecutive patients undergoing RFCA of OT-VPCs were divided into two groups according to the outcomes of ablation. There were no significant differences regarding gender, age, disease, symptomatic status, a history of drinking, hypertension, diabetes, or based on LVEF, LVEDD, LVESD, E/E', and E/A between patients with and without initially successful ablation (Table 1).

### 3.2. Results of Catheter Ablation

Initially successful elimination of OT-VPCs was achieved in 136 patients, while failed RFCA was recorded for 18 patients. The acute success rate was 88.3% (136/154).

When repeated and careful ablation was performed at the relative ideal targeted areas (confirmed by a mapping system) where the ablation catheter could be positioned, the VPCs could be eliminated during the ablation procedure, but resume after stopping radiofrequency energy. It was considered that a prolonged ablation duration would not improve the procedure's success rate and may even have increased the risk of complications because of the deeper origin of the VPCs. Thus, the procedure was stopped in 18 patients, giving rise to a failed ablation. Among these 18 patients, two VPCs could not be mapped because the catheter was obstructed in the interior of the coronary sinus. Furthermore, two VPCs were adjacent to the coronary artery opening, which presented a higher risk of acute coronary occlusion for long-term ablation. One VPC failed because of intolerable chest pain experienced by the patient during the ablation. One VPC originated from the distal end of the great cardiac vein, where the impedance was greater than 300 ohms; as a result, the discharge could not effectively continue.

The origin of VPCs in 154 patients was recorded as follows. For 118 patients, this was at the RVOT, at the left ventricular OT (LVOT) for 31 patients, and for five patients, the origin occurred at both. For the patients that experienced failed ablation, the origin site of VPCs was from the RVOT septum (*N* = 5), the junction of the RVOT septum and the RVOT free wall (*N* = 2), the left sinus of the pulmonary artery (*N* = 1), the left coronary sinus (*N* = 1), the aortomitral continuity (AMC) area (*N* = 2), the great cardiac vein (*N* = 2), and the summit area (*N* = 2). Thus, for patients who experienced a procedural failure, eight were derived from the RVOT, seven from the LVOT, and three from both locations. In total, 23 of 154 patients had pleomorphic VPCs; 20 of these 23 patients were found in the successful group, while the remaining three were in the other group.

A chi-square test and Fisher's exact test demonstrated a significant difference in the origin of VPCs between the successful and the failed groups. The highest success rate was in patients with VPCs from the RVOT (110/118), and the lowest success rate was in patients with VPCs from both the RVOT and LVOT (2/5). There was, however, no difference when patients with and without pleomorphic VPCs were compared with respect to initial outcomes (Table 2).

### 3.3. Electrocardiographic Features

The 136 patients with successful procedures had a lower ratio showing the existence of a pseudo *Δ*wave (16.2% vs. 44.4%, *P* < 0.01), a smaller R-wave amplitude in lead V1 (0.23 ± 0.24 mV vs. 0.35 ± 0.44 mV, *P* < 0.05), a shorter IDT in lead V2 (44.00 ± 18.33 ms vs. 57.41 ± 20.67 ms, *P* < 0.01), a shorter RS duration in lead V2 (93.67 ± 21.33 ms vs. 106.93 ± 18.76 ms, *P* < 0.01), and a smaller R/S ratio in lead V2 (0.21 ± 0.32 vs. 0.37 ± 0.90, *P* < 0.05). There was no significant difference in the interpolated VPCs; the QRS ugly sign; the retrograde P-wave; the QTc interval; the QRS duration in leads II, III, aVF, and V2; the R-wave amplitude in leads II, III, aVF, and V2; the R-wave ratio of leads III/II; the S-wave amplitude in lead V2; the Q-wave amplitude in leads aVL and aVR; the Q-wave ratio of leads aVL/aVR; or in the MDI between patients with and without failed ablation (Table 3).

### 3.4. Predictors of Failed Ablation in Multivariate Analysis

Based on multivariate analysis, we selected the variables VPC origin, pleomorphic VPCs, pseudo delta wave, R-wave amplitude in V1, IDT in V2, RS duration in V2, and R/S ratio in V2 for further analysis. Binary logistic regression analysis demonstrated RS duration, and R/S ratio in V2 could independently predict a failed procedure (*P* = 0.029, odds ratio [OR]: 1.044, 95% confidence interval [CI]: 1.005–1.0865; *P* = 0.041, OR: 1.890, 95% CI: 1.028–3.484; see Table 4).

**Figure 2:**
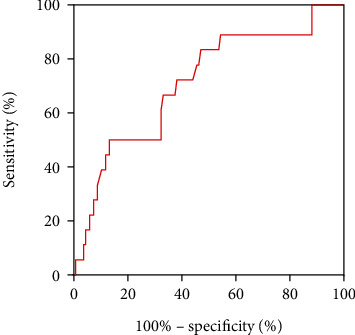
The receiver-operator characteristic analysis of RS duration in lead V2.

**Figure 3:**
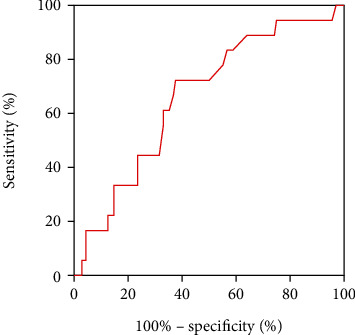
The receiver-operator characteristic analysis of the R/S ratio in lead V2.

Based on the ROC curve analysis, cut-off values for RS duration in V2 that were larger than 109.17 ms and R/S ratio in V2 that was larger than 0.28 could predict failed ablation (sensitivity =50% and specificity =86.8%; sensitivity =72.2% and specificity =62.5%, respectively; see Figures 2 and 3, and Table 5).

### 3.5. Prognosis and Complications

During a mean follow-up period of 12.5 months, eight patients (5.9%) had recurrences after initially successful ablation. Among them, seven patients accepted secondary RFCA and had a 100% successful ablation rate. For two patients, the origin area of VPC changed. None of the 154 patients died. One patient developed ventricular fibrillation during ablation, which was restored to a sinus rhythm after electrical defibrillation; two patients developed a prolonged P-R interval after ablation. No other acute or long-term complications were observed.

## 4. Discussion

Recent evidence suggested that RFCA could play a safer and more effective role in the treatment of patients with OT-VPCs. Despite advancements in electro-anatomical mapping and ablation technology and a high acute success rate of approximately 80%–90% in general, variable recurrence and failure rates have been reported. Therefore, the need for strengthening the anatomical structure related to the site of the origin of VPCs, ablation catheter selection, the approach for ablation, and whether a catheter can reach the designated position is needed to improve the success rate and reduce recurrence.

### 4.1. The Anatomical Structure

Alongside a deepening understanding of cardiac anatomy, the concept of the cardiac summit region, also known as the left ventricular summit (LVS), was put forward. The LVS is located in the epicardial triangle outside the left ventricular outlet and can generally be divided into epicardial and endocardial parts.

The epicardial part is a triangular portion of the epicardial LVOT and is bounded by bifurcation between the left anterior descending and the left circumflex coronary arteries; it is transected laterally by the great cardiac vein (GCV) at its junction with the anterior interventricular vein (AIV), which is also known as the GCV/AIV junction. The ablation of VPCs originating from the GCV/AIV junction is difficult because it is partially covered by a thick layer of epicardial fat and lies adjacent to major blood vessels.

Conversely, the endocardial part of the LVS includes the AMC, the left coronary cusp (LCC), and the posterior septum of the right ventricular OT (RVOT). The AMC region is a triangular fibrous structure formed by the aortic sinus and the anterior lobe of the mitral valve, while the LCC is adjacent to both the left ventricular epicardium and the endocardium [7, 8].

### 4.2. Predictors of the Success of Ablation and Recurrence

An international large-scale, multicenter analysis of the outcomes of the ablation of idiopathic VPCs suggested that VPCs originated from the epicardium or that multiple locations were predictive of procedural failure; patients with VPCs that originated in the RVOT reflected the highest success rate and a low complication rate, and the only significant predictor for long-term procedural success post-ablation was an RVOT VPC origin [9]. The possible reasons for patients experiencing the above-mentioned failed RFCA outcomes are as follows: (1) the epicardium has a more complex anatomical structure and is close to important blood vessels, which require a relatively lower ablation power and shorter procedure duration, and (2) pleomorphic VPCs typically originate from the epicardium and papillary muscles, where ablation may take longer to complete [10].

Contrary to the above, Baser et al. [11] found no significant difference in the acute success rate of ablation among patients with or without pleomorphic VPCs, contrary to the success rate at three months post-ablation. The study also indicated no site of origin as being predictive of the outcome at three months. This was likely because the procedure had been defined as acutely effective by them if the predominant VPC had been eliminated and did not recur for 12-h post-ablation. For patients with pleomorphic VPCs, even when it was impossible to completely eliminate all the VPCs, patients could still benefit from the ablation through the successful elimination of the predominant VPCs and have symptomatic improvement. Thus, the acute success rate of ablation in pleomorphic VPCs increased, while the long-term procedural success post-ablation decreased because the nonpredominant VPC could possibly become the predominant VPC during follow-up.

Chung et al. [12] put forward the concept of early and late recurrences post-ablation, and that patients with early and late recurrences demonstrated nonuniform patterns of clinical characteristics and electrophysiological properties. Patients with hypertension were more likely to experience a late recurrence; however, the pathogenesis for this result remained unclear. It may have been linked to patients with refractory hypertension who became anxious or easily stressed. However, no relevant literature has been reported to confirm whether the effective control of blood pressure could reduce the recurrence of VPCs.

Our study suggests that the initial success rate of 93.2% (110/118) for RFCA in patients with RVOT-VPCs was significantly higher compared with the 77.4% (24/31) for RFCA among patients with LVOT-VPCs, which is consistent with the foregoing. In fact, some of arrhythmias classified as LVOT or both LVOT/RVOT in our study could actually have been originating from LV summit. The efficacy of ablation drops significantly if multisite ablation of LV summit VPC is required [13]. In addition, we found that among patients with failed ablation of VPCs originating from the RVOT, seven cases were mapped and ablated under the pulmonary valve, while one was ablated on the pulmonary valve [14]. Of the 110 initial successful cases of RVOT-VPCs ablation, 70 were all mapped and ablated on the pulmonary valve, and 38 were mapped and ablated under the pulmonary valve. The success rate of ablation on the pulmonary valve was much higher compared with the procedure under the pulmonary valve (98.6% vs. 84.4%, *P* < 0.01). This confirmed that with the continued development of RFCA technology, the primary ablation strategy selection for RVOT-VPCs was to target the pulmonary artery sinus rather than the pulmonary valve.With a change in this ablation strategy, the success rate of ablation for patients with RVOT-VPCs had been significantly improved; however, this may have led to a bias in the ablation results observed in this study. There was no guarantee that patients for whom ablation was targeted at the septal part or at the free wall of the RVOT would experience the same outcome if mapping and ablation on the pulmonary valve had been performed. This represents another limitation in our study.

Although our study indicated no significant guidelines for the forecasting of successful ablation in patients with and without pleomorphic VPCs, careful analysis confirmed that in all of the patients with pleomorphic VPCs who had successful ablation, the multiple origins of VPCs were further away from different parts of the same OT (18/20). Only two cases originated from different parts of the different OTs (2/20) and, among the patients that experienced failed ablation, the multiple origins of VPCs all derived from different parts of the different OTs (3/3). Accordingly, for patients with pleomorphic VPCs that originated from different OTs, the success rate was only 40% (2/5); this also confirmed the results mentioned above from the side.

### 4.3. Electrocardiographic Predictors of Radiofrequency Ablation Outcomes

Existing studies have reported ECG predictors of RF ablation outcomes. Vestal et al. considered the presence of QRS morphologic variation, a wider mean QRS width, and a taller mean R-wave amplitude in lead II as ECG predictors for failed RVOT VPC ablation; the only ECG predictor of recurrence was the presence of RVOT VPC QRS morphological variation [4]. A QRS morphological shift following RFCA could be observed in 4% of patients with idiopathic OT-VPCs, which indicated that detailed remapping was necessary; this was because the successful ablation site for the VPCs with an altered QRS morphology shifted to different anatomical structures in most patients. Concurrently, following successful ablation for VPCs by remapping the best target, the presence of a subsequent QRS morphological shift indicated recurrence [15]. On the one hand, the longer QRS duration prompted the ventricular ectopic rhythm point to deflect from the normal cardiac conduction system, which indicated difficulty placing the ablation catheter in a manner that enabled it to reach the ablation target site. On the other hand, it also predicted the proliferation and fibrosis of myocardial cells and a conduction delay of electrical impulses in the myocardial cells, thus resulting in ablation failure. The taller R-wave amplitude, particularly in lead II, suggested an ectopic rhythm point located closer to the top of the ventricles, which is typically covered with tough fibrous tissue, thereby increasing the difficulty of the ablation.

Hachiya et al. demonstrated that a peak deflection index (PDI) higher than 0.6 was associated with a higher rate of failed ablation, which suggested that VPCs originated from deep within the ventricular septum or epicardium, thereby affecting the ablation results [16].

Tanner et al. [17] reported that a V2 R-wave amplitude larger than 30% of the QRS amplitude predicted a failed ablation, and someone else reported a Q-wave ratio of leads aVL/aVR higher than 1.4, an R-wave ratio of leads III/II higher than 1.1, and a higher R/S amplitude ratio of lead V1 as being associated with a higher incidence of unsuccessful RFCA.

The ECG features of failed RVOT ablation were characterized by a PDI higher than 0.57, a V2 R-wave duration higher than 44 ms, and a V2 transition ratio above 0.73 by Yamada et al. [6]. The longer R-wave duration and the larger transition ratio in lead V2 suggested that the origin of VPCs was close to the aortic valve ring.

Sriratanasathavorn et al. [18] demonstrated that the monophasic R-wave in lead I during RVOT tachycardia was associated with unfavorable outcomes after RF ablation and may reflect the origin of VPCs as being from the right ventricle free wall site. When compared with septal sites, free wall sites more often have a structural disease, such as localized wall bulging, wall thinning, fatty infiltration, and fibrosis, which can lead to recurrent tachycardia in these patients.

Our study indicated that RS duration in lead V2 and R/S ratio in lead V2 were independent predictors for a low RFCA success rate in patients with VPCs. In addition, the QRS duration in leads II, III, aVF, and V2; the R-wave amplitude in leads II, III, aVF, and V2; the R-wave ratio of leads III/II; the S-wave amplitude in lead V2; the Q-wave ratio of leads aVL/aVR; and MDI could not predict the results. The pseudo*Δ*wave, a taller R-wave amplitude in lead V1, and a longer IDT in lead V2 were able to predict a failed procedure with weak reliability.

The V2 lead is a precordial lead located at the upper left of the RVOT, directly opposite the LVOT. Anatomically, the LVOT is located in the left posterior of RVOT and thus showed a wider and higher R-wave pattern in V2 lead compared with patients with RVOT-VPCs. Therefore, the longer RS duration and the larger R/S ratio in V2 meant the origin areas were closer to the LVOT and extended beyond the LVS, which lead to ablation difficulties [8, 19–21]. This is fundamentally consistent with the information noted above related to the origin site of VPCs affecting the ablation success rate.

Our study proposes two ECG parameters that could enable faster data results without complex computing and avoid individual detection errors as much as possible. This outcome was based on the placement of lead V2 being relatively fixed at the parasternal second intercostal space compared with other leads. However, our study presents the problem that the sensitivity and the specificity of the two parameters were not sufficient. Accordingly, further studies are needed to find more satisfying ECG parameters.

### 4.4. Limitations

The present study includes several limitations. First, the research sample was small, particularly the number of cases in the failure group. More and larger samples are needed to strengthen the credibility of our research results. Second, owing to the retrospective nature of the study, some of the patients could not be included in the research due to a lack of clinical data. Third, RFCA technology continues to improve, as does the experience and abilities of doctors, which may lead to bias in research results. Fourth, due to differences in individual body shapes (such as height, weight, and thoracic shape), differences in environment, and differences in the placement of leads, individual detection errors in ECG exams may have occurred. Fifth, although all ECG parameters were measured by the same person, human measurement errors could not be excluded. Sixth, in spite of the predictive value of the two ECG parameters presented herein, their sensitivity and specificity were not sufficient; therefore, additional research is needed to establish ECG parameters that are satisfactory in this regard.

## 5. Conclusions

The efficacy and safety of catheter radiofrequency ablation for OT-VPCs were described in this study. The ECG predictors of failed ablation were characterized by RS duration and R/S ratio in lead V2, and an RS duration in lead V2 above 109.17 ms; a DR/S ratio in lead V2 above 0.28 was associated with a lower rate of successful ablation.

## Figures and Tables

**Table 1:** Comparison of clinical characteristics in patients with initially successful and failed RFCA (*N* = 154).

	Initially success *N* = 136	Failure *N* = 18	*P* value
Male (%)	34 (25.0%)	8 (44.4%)	0.083
Age (years)	48.28 ± 13.20	56.00 ± 26.00	0.311
Course (months)	12.00	9.00	0.451
Presence of symptoms (%)	134 (98.5%)	17 (94.4%)	0.313
History of smoking (%)	15 (11.0%)	4 (22.2%)	0.242
History of drinking (%)	7 (5.1%)	2 (11.1)	0.600
Hypertension (%)	32(23.5%)	7(38.9%)	0.162
Diabetes mellitus (%)	9 (6.6%)	3 (16.7%)	0.150
LVEF (%)	65.13 ± 5.86	65.13 ± 5.86	0.297
LVEDD (millimeter)	48.80 ± 4.46	50.78 ± 3.32	0.198
LVESD (millimeter)	30.80 ± 4.90	32.71 ± 2.50	0.095
E/E'	10.00 ± 3.70	11.65 ± 4.13	0.585
E/A	1.10 ± 0.50	1.01 ± 0.42	0.262

**Table 2:** Comparison of VPC Origin and pleomorphic VPCs in patients with initially successful and failed RFCA (*N* = 154).

	Initially success *N* = 136	Failure *N* = 18	*P* value
VPC origin			0.001∗∗
RVOT (%)	110 (80.9%)	8 (44.4%)	
LVOT (%)	24 (17.6%)	7 (38.9%)	
Both (%)	2 (1.5%)	3 (16.7%)	
Pleomorphism (%)	20 (14.7%)	3 (16.7%)	0.734

∗∗*P* < 0.01, RVOT: right ventricular outflow tract; LVOT: light ventricular outflow tract.

**Table 3:** Comparison of ECG parameters in patients with initially successful and failed RFCA (*N* = 154).

	Initially success *N* = 136	Failure *N* = 18	*P* value
Interpolated VPCs (%)	23 (16.9%)	1 (5.6%)	0.310
Retrograde P-wave (%)	35 (25.7%)	4 (22.2%)	1
QRS ugly sign (%)	11 (8.1%)	2 (11.1%)	0.651
Pseudo delta wave (%)	22 (16.2%)	8 (44.4%)	0.009∗∗
QTc interval (ms)	430.38 ± 26.56	435.50 ± 28.25	0.978
QRS duration in lead II (ms)	151.51 ± 14.62	154.50 ± 22.83	0.068
R wave amplitude in lead II (mV)	1.88 ± 0.48	2.03 ± 0.40	0.290
QRS duration in lead III (ms)	148.33 ± 14.47	152.39 ± 18.51	0.219
R wave amplitude in lead III (mV)	1.80 ± 0.57	2.15 ± 0.74	0.209
QRS duration in lead aVF (ms)	149.12 ± 14.54	152.33 ± 35.42	0.081
R wave amplitude in lead aVF (mV)	1.82 ± 0.52	1.97 ± 0.53	0.339
Q wave amplitude in lead aVR (mV)	0.92 ± 0.22	1.00 ± 0.22	0.280
Q wave amplitude in lead aVL (mV)	0.81 ± 0.37	0.96 ± 0.41	0.333
R-wave ratio of leads III/II	0.98 ± 0.20	1.00 ± 0.24	0.715
Q-wave ratio of leads aVL/aVR	0.91 ± 0.43	0.91 ± 0.51	0.980
MDI of inferior lead	0.57 ± 0.08	0.58 ± 0.09	0.512
R wave amplitude in V1 (mV)	0.23 ± 0.24	0.35 ± 0.44	0.036∗
IDT in lead V2 (ms)	44.00 ± 18.33	57.41 ± 20.67	0.005∗∗
RS duration in V2 (ms)	93.67 ± 21.33	106.93 ± 18.76	0.004∗∗
QRS duration in V2 (ms)	147.82 ± 17.95	153.11 ± 16.61	0.205
R wave amplitude in V2 (mV)	0.42 ± 0.49	0.71 ± 0.58	0.056
S wave amplitude in V2 (mV)	1.94 ± 0.91	1.61 ± 1.04	0.145
R/S ratio in lead V2	0.21 ± 0.32	0.37 ± 0.90	0.032∗

∗*P* < 0.05, ∗∗*P* < 0.01.

**Table 4:** Multivariate analysis of variables in prediction of failed ablation of VPCs.

	*P* value	OR value	95% CI
RS duration in V2	0.029	1.044	1.005-1.086
R/S ratio in V2	0.041	1.890	1.028-3.484

**Table 5:** ROC analysis of RS duration in V2 and R/S ratio in V2.

	Area	Standard error	*P* value	95% CI
RS duration in V2	71%	0.067	0.004	0.579-0.840
R/S ratio in V2	65.6%	0.065	0.032	0.529-0.783

## Data Availability

The datasets used and/or analyzed during the current study available from the corresponding author on reasonable request.

## References

[1] Dukes J. W., Dewland T. A., Vittinghoff E. (2015). Ventricular ectopy as a predictor of heart failure and death. *Journal of the American College of Cardiology*.

[2] Dabbagh G. S., Bogun F. (2017). Predictors and therapy of cardiomyopathy caused by frequent ventricular ectopy. *Current Cardiology Reports*.

[3] Stec S., Sikorska A., Zaborska B., Kryński T., Szymot J., Kułakowski P. (2012). Benign symptomatic premature ventricular complexes: short- and long-term efficacy of antiarrhythmic drugs and radiofrequency ablation. *Kardiologia Polska*.

[4] Ling Z., Liu Z., Su L. (2014). Radiofrequency ablation versus antiarrhythmic medication for treatment of ventricular premature beats from the right ventricular outflow tract: prospective randomized study. *Circulation Arrhythmia and Electrophysiology*.

[5] Vestal M., Wen M. S., Yeh S. J., Wang C. C., Lin F. C., Wu D. (2003). Electrocardiographic predictors of failure and recurrence in patients with idiopathic right ventricular outflow tract tachycardia and ectopy who underwent radiofrequency catheter ablation. *Journal of Electrocardiology*.

[6] Yamada S., Chung F. P., Lin Y. J. (2018). Electrocardiographic features of failed and recurrent right ventricular outflow tract catheter ablation of idiopathic ventricular arrhythmias. *Journal of Cardiovascular Electrophysiology*.

[7] Lin C. Y., Chang S. L., Lin Y. J. (2015). Long-term outcome of multiform premature ventricular complexes in structurally normal heart. *International Journal of Cardiology*.

[8] Yamada T., Litovsky S. H., Kay G. N. (2008). The left ventricular Ostium. *Circulation Arrhythmia and Electrophysiology*.

[9] Latchamsetty R., Yokokawa M., Morady F. (2015). Multicenter outcomes for catheter ablation of idiopathic premature ventricular complexes. *JACC: Clinical Electrophysiology*.

[10] Sheldon S. H., Latchamsetty R., Morady F., Bogun F. (2017). Catheter ablation in patients with pleomorphic, idiopathic, premature ventricular complexes. *Heart Rhythm*.

[11] Baser K., Bas H. D., Belardi D. (2014). Predictors of outcome after catheter ablation of premature ventricular complexes. *Journal of Cardiovascular Electrophysiology*.

[12] Chung F. P., Chong E., Lin Y. J. (2014). Different characteristics and electrophysiological properties between early and late recurrences after acute successful catheter ablation of idiopathic right ventricular outflow tract arrhythmias during long-term follow-up. *Heart Rhythm*.

[13] Chung F. P., Lin C. Y., Shirai Y. (2020). Outcomes of catheter ablation of ventricular arrhythmia originating from the left ventricular summit: a multicenter study. *Heart Rhythm*.

[14] Deng C. G., Zhang J. L., Li Z. (2018). A new idea and approach for the treatment of idiopathic ventricular arrhythmia of right ventricular outflow tract: mapping and ablation of pulmonary sinus. *Zhong Hua Xin Lv Shi Chang Za Zhi*.

[15] Shirai Y., Liang J. J., Garcia F. C. (2018). QRS morphology shift following catheter ablation of idiopathic outflow tract ventricular arrhythmias: prevalence, mapping features, and ablation outcomes. *Journal of Cardiovascular Electrophysiology*.

[16] Hachiya H., Hirao K., Sasaki T. (2010). Novel ECG predictor of difficult cases of outflow tract ventricular tachycardia: peak deflection index on an inferior lead. *Circulation Journal*.

[17] Tanner H., Wolber T., Schwick N., Fuhrer J., Delacretaz E. (2004). Electrocardiographic pattern as a guide for management and radiofrequency ablation of idiopathic ventricular tachycardia. *Cardiology*.

[18] Krittayaphong R., Sriratanasathavorn C., Dumavibhat C. (2006). Electrocardiographic predictors of long-term outcomes after radiofrequency ablation in patients with right-ventricular outflow tract tachycardia. *Europace*.

[19] Hamon D., Blaye-Felice M. S., Bradfield J. S. (2016). A new combined parameter to predict premature ventricular complexes induced cardiomyopathy: impact and recognition of epicardial origin. *Journal of Cardiovascular Electrophysiology*.

[20] Ouyang F., Fotuhi P., Ho S. Y. (2002). Repetitive monomorphic ventricular tachycardia originating from the aortic sinus cusp: electrocardiographic characterization for guiding catheter ablation. *Journal of the American College of Cardiology*.

[21] Ito S., Tada H., Naito S. (2003). Development and validation of an ECG algorithm for identifying the optimal ablation site for idiopathic ventricular outflow tract tachycardia. *Journal of Cardiovascular Electrophysiology*.

